# Machine learning for predicting post-operative outcomes in meningiomas: a systematic review and meta-analysis

**DOI:** 10.1007/s00701-024-06344-z

**Published:** 2024-12-17

**Authors:** Siraj Y. Abualnaja, James S. Morris, Hamza Rashid, William H. Cook, Adel E. Helmy

**Affiliations:** 1 Peterborough City Hospital, Peterborough, UK; 2 University of Cambridge, Cambridge, UK; 3 Division of Neurosurgery, Department of Clinical Neurosciences, University of Cambridge, Cambridge, UK

**Keywords:** Meningioma, Machine learning, Postoperative outcomes, Predictive accuracy, Survival prognosis

## Abstract

**Purpose:**

Meningiomas are the most common primary brain tumour and account for over one-third of cases. Traditionally, estimations of morbidity and mortality following surgical resection have depended on subjective assessments of various factors, including tumour volume, location, WHO grade, extent of resection (Simpson grade) and pre-existing co-morbidities, an approach fraught with subjective variability. This systematic review and meta-analysis seeks to evaluate the efficacy with which machine learning (ML) algorithms predict post-operative outcomes in meningioma patients.

**Methods:**

A literature search was conducted in December 2023 by two independent reviewers through PubMed, DARE, Cochrane Library and SCOPUS electronic databases. Random-effects meta-analysis was conducted.

**Results:**

Systematic searches yielded 32 studies, comprising 142,459 patients and 139,043 meningiomas. Random-effects meta-analysis sought to generate restricted maximum-likelihood estimates for the accuracy of alternate ML algorithms in predicting several postoperative outcomes. ML models incorporating both clinical and radiomic data significantly outperformed models utilizing either data type alone as well as traditional methods. Pooled estimates for the AUCs achieved by different ML algorithms ranged from 0.74–0.81 in the prediction of overall survival and progression-/recurrence-free survival, with ensemble classifiers demonstrating particular promise for future clinical application. Additionally, current ML models may exhibit a bias in predictive accuracy towards female patients, presumably due to the higher prevalence of meningiomas in females.

**Conclusion:**

This review underscores the potential of ML to improve the accuracy of prognoses for meningioma patients and provides insight into which model classes offer the greatest potential for predicting survival outcomes. However, future research will have to directly compare standardized ML methodologies to traditional approaches in large-scale, prospective studies, before their clinical utility can be confidently validated.

**Supplementary Information:**

The online version contains supplementary material available at 10.1007/s00701-024-06344-z.

## Background

Meningioma is the most common primary brain tumour and accounts for more than one-third of all cases [21]. According to the World Health Organization's (WHO) 2021 Classification of CNS tumours, meningiomas are categorised into three grades (1–3) based on histological and molecular criteria, with the grade directly influencing prognosis [21, 40]. While diagnosis is ultimately confirmed histologically, initial assessments commonly rely on MRI and CT imaging not only to suggest a provisional diagnosis but also to guide our approach to surgical resection. The latter aims to achieve maximal reduction in tumour while preserving neurological function [21, 40] and forms the mainstay of treatment, particularly for symptomatic or rapidly growing meningiomas. However, surgical intervention carries risks of complications which may significantly impact patient quality of life [40].

Traditionally, estimations of morbidity and mortality following resection of the meningioma have depended on clinicians’ assessments of various factors, including tumour volume, location, WHO grade, extent of resection (Simpson grade) and pre-existing co-morbidities, an approach fraught with subjective variability [22, 40]. This uncertainty is a significant source of anxiety for patients considering surgery and underscores the need for more precise, quantifiable risk evaluations.

Machine learning (ML) algorithms represent a paradigm shift in this context, providing a refined means of projecting outcomes such as recurrence, survival, and neurological integrity, hence delivering a relatively unbiased prognostic tool. A previous meta-analysis has already demonstrated the commendable accuracy of ML algorithms in diagnosing meningioma using radiomic data [22]. However, their accuracy in predicting post-surgical outcomes has not yet been assessed through meta-analytical methods. Accordingly, this paper seeks to review the current literature to generate pooled estimates for the overall accuracy of different classes of ML algorithms in predicting postoperative outcomes in meningioma patients.

## Methods

### Protocol and registration

This review and meta-analysis was conducted according to a prospective protocol, registered with the Prospective Register of Systematic Reviews (PROSPERO) in order to limit bias (CRD42023475857).

### Literature search and eligibility criteria

A literature search was conducted in December 2023 by two independent reviewers, SA and JM, through PubMed, DARE, Cochrane Library and SCOPUS electronic databases. Systematic searches were limited to articles published between 1980–2023 and utilized the following search parameters: “Meningioma” AND “ML” AND “Outcome Prediction”; “Meningioma” AND “Predictive Models” AND “Outcome Prediction”; “Meningioma” AND “ML” AND “Predictive Models”; “Meningioma” AND “ML” AND “Prognosis”; “Meningioma” AND “Predictive Models” AND “Prognosis”. Each paper returned was screened using the full title and abstract, before scrutinizing full-text articles for all studies that remained. Disputes were resolved by discussion with a third party (HR).

### Inclusion criteria

Article screening sought to include original research articles published in peer-reviewed journals between 1980 and 2023. Both retrospective and prospective studies were included provided they reported the efficacy with which any ML algorithm predicted postoperative outcomes in patients with meningioma. Particular attention was paid to the main outcomes of interest in this review, namely overall survival, progression-free survival and recurrence-free survival. Randomised controlled trials, case–control studies and cohort studies were all considered for inclusion.

### Exclusion

Review articles, case series, case reports and conference abstracts were all excluded, as were papers for which a full-text article was unavailable in English. We also excluded studies conducted on non-human animals, or which did not provide a quantitative measure of the predictive efficacy with regards to postoperative outcomes.

### Quality assessment and risk of bias

The quality of included studies was assessed using the Newcastle–Ottawa Scale before trichotomizing them according to the standards of the Agency for Healthcare Research and Quality (AHRQ). Those included in the meta-analysis were evaluated for publication bias, both through visual inspection of the funnel plot and by objective interpretation of the Egger’s test, using a significance threshold of α = 0.05.

### Data extraction

For each outcome and each class of ML algorithm, the area under the receiver operating characteristic (ROC) curve (AUC) was recorded along with its 95% confidence interval. Where AUC was not explicitly reported but instead presented in graphical format, it was estimated using the number of pixels under the curve. Additionally, data regarding patient demographics and study characteristics in each paper were extracted for use in meta regressions.

### Statistical analysis

Random-effects meta-analyses were conducted to generate restricted maximum-likelihood estimates for the AUC of each outcome and each class of ML algorithm assessed. For each estimate, the between-study heterogeneity was assessed via I^2^ values, interpreted using predefined thresholds for low, moderate and high heterogeneity of < 25%, 25–75% and > 75% [12]. Meta-regressions were conducted using the log-transformed AUC against each of three study-level covariates to identify any correlations between these covariates and the efficacy of ML algorithms. These covariates were the mean age and the sex ratio of patients in each study as well as the proportion of patients who underwent total tumour resection (defined as Simpson Grade ≤ 3). The validity of the linear model was confirmed by visual inspection of the plotted residuals and each covariate significantly correlated with ln(AUC) was assessed for collinearity via auxiliary regressions between each pair of significant covariates. Additionally, variation inflation factors (VIF) were also used to detect collinearity between covariates and identify any potential confounding between the covariates used in meta-regressions [2]. Statistics are presented in the format [estimate] ([95% confidence interval]) unless otherwise stated. All statistical analyses were conducted by JM using STATA v18.0 (StataCorp LLC, Texas).

## Results

### Systematic searches

**Fig. 1:**
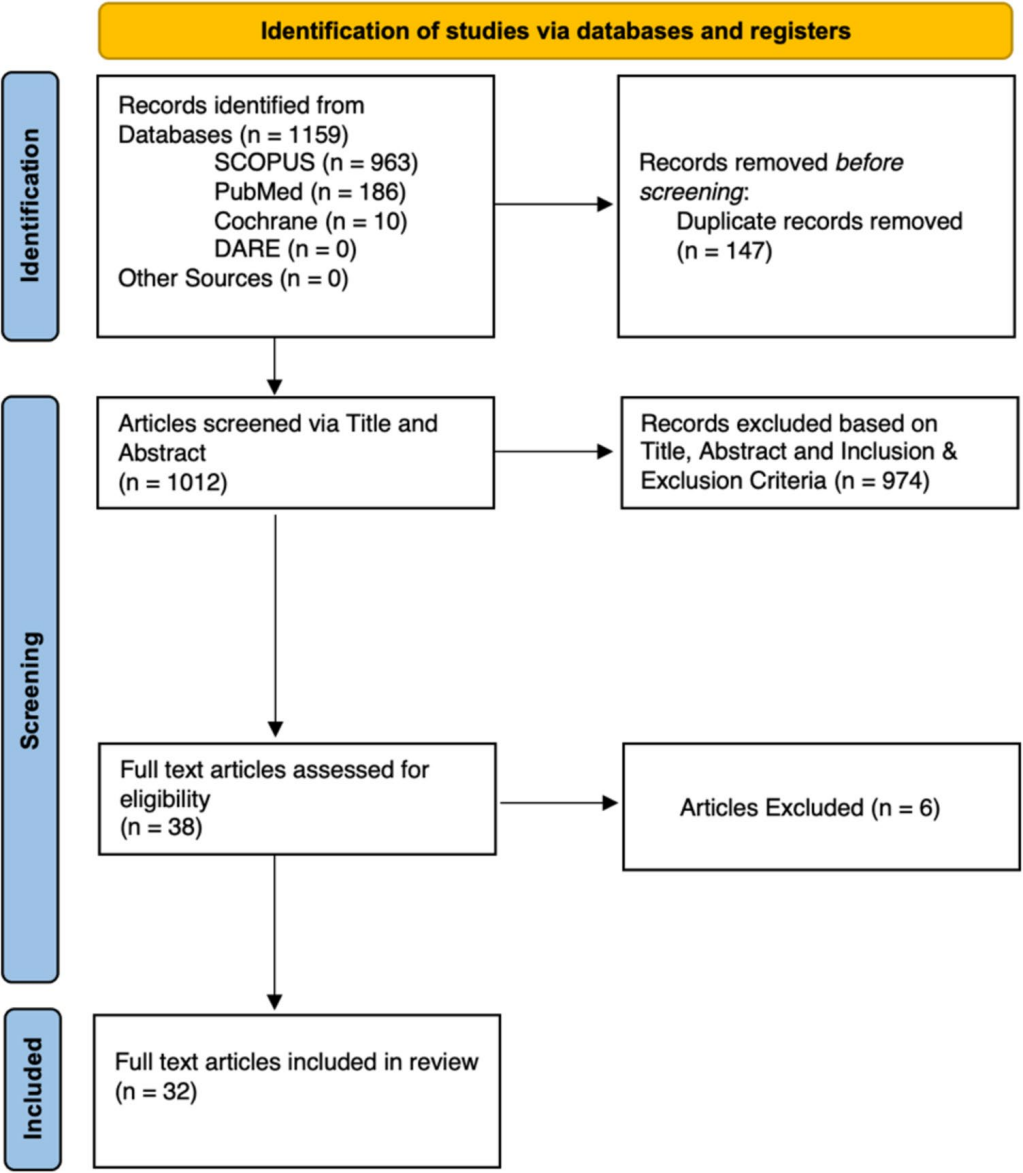
PRISMA Diagram representing the inclusion and exclusion of the studies selected for this review

Systematic searches of SCOPUS, PubMed, Cochrane Library and DARE yielded 963, 186, 10 and 0 references, respectively, of which, 147 were duplicates. 974 were excluded after title and abstract screening and a further 6 were excluded upon full-text review, leaving a total of 32 unique studies in this review (Fig. 1 , Supplementary Table 1).

### Study characteristics

A total of 32 studies (Supplementary Table 1) involving 142,459 individual patients and 139,043 meningiomas were included in this review. The majority were single-centre, retrospective cohort studies, while the four largest studies accessed national databases of ≥ 5,000 patients. The latter contributed a total of 127,020 (89.2%) patients included in this review. However, there was considerable overlap between the cohorts of several studies; for example, all 303 meningioma patients included in one multi-database study [6] also accounted for the entire cohort from another included study published the following year [25]. Likewise, two papers selected their meningioma patient data from the same source, the National Inpatient Sample [27, 42]. These papers covered a time frame of 11 years, 3 of which overlapped, thus there may be a slight bias in our pooled estimates towards the characteristics of this subgroup of patients. For papers in which the cohort completely overlapped with that of another paper, the smaller of the two was excluded. Overall, the mean age of the patients in our analysis was 56.03 (range 5–99) while the M:F ratio was 37:63, in keeping with the known sex ratio of meningiomas. Where reported, 43.5% of meningiomas were found in the skull base and 71.8% were managed by total excision (Simpson grade ≤ 3). 31 of the included studies were published in the seven years since 2017 and all but one exclusively recruited meningioma patients [6].

### Data utilisation and model types

**Fig. 2:**
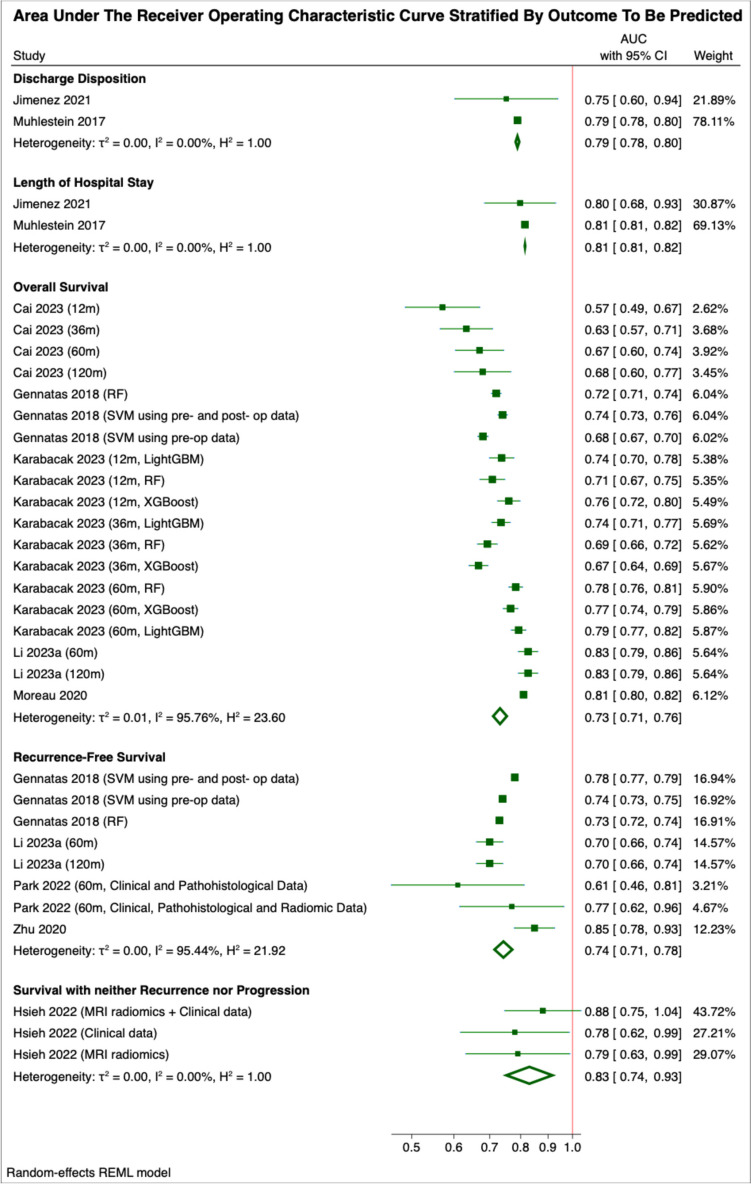
Area under the receiver operating characteristic curve stratified by the outcome to be predicted using ML algorithms. Weights are given with respect to each outcome. Where the same paper is included multiple times, each estimate is discriminated by the time course of outcome prediction in months, the ML algorithm employed or the modality of the input data

The numbers of studies reporting the predictive accuracy of each class of ML algorithm were as follows: Logistic Regression (*n* = 20) [4, 6–10, 13, 14, 16, 24, 26–30, 38, 39, 41, 44, 45], Random Forests (*n* = 16) [6, 8, 11, 14–16, 19, 23–28, 31, 34, 43], Support Vector Machines (*n* = 12) [6, 8, 11, 16–18, 23, 26, 27, 31, 34, 41], Cox Proportional-Hazards Regression (*n* = 4) [3, 5, 37, 42], Decision Trees (*n* = 8) [6, 8, 11, 16, 20, 26, 28, 30], Neural Networks (*n* = 1) [29], Gradient Boosting (*n* = 7) [6, 8, 11, 15, 16, 26, 34] and Step-Wise Regression (*n* = 1) [2]. Ensemble algorithms, which integrate several ML approaches in order to generate more robust predictions, were examined in five studies (*n* = 5) [11, 23, 24, 26, 27]. The numbers of studies investigating each postoperative outcome were: recurrence-free survival (*n* = 12), overall survival (*n* = 8), specific complications (*n* = 5), discharge disposition (*n* = 4), progression-free survival (*n* = 3), survival with neither recurrence nor progression (*n* = 3), and length of hospital stay (*n* = 2) (Fig. 2).

### Summary of results

**Fig. 3:**
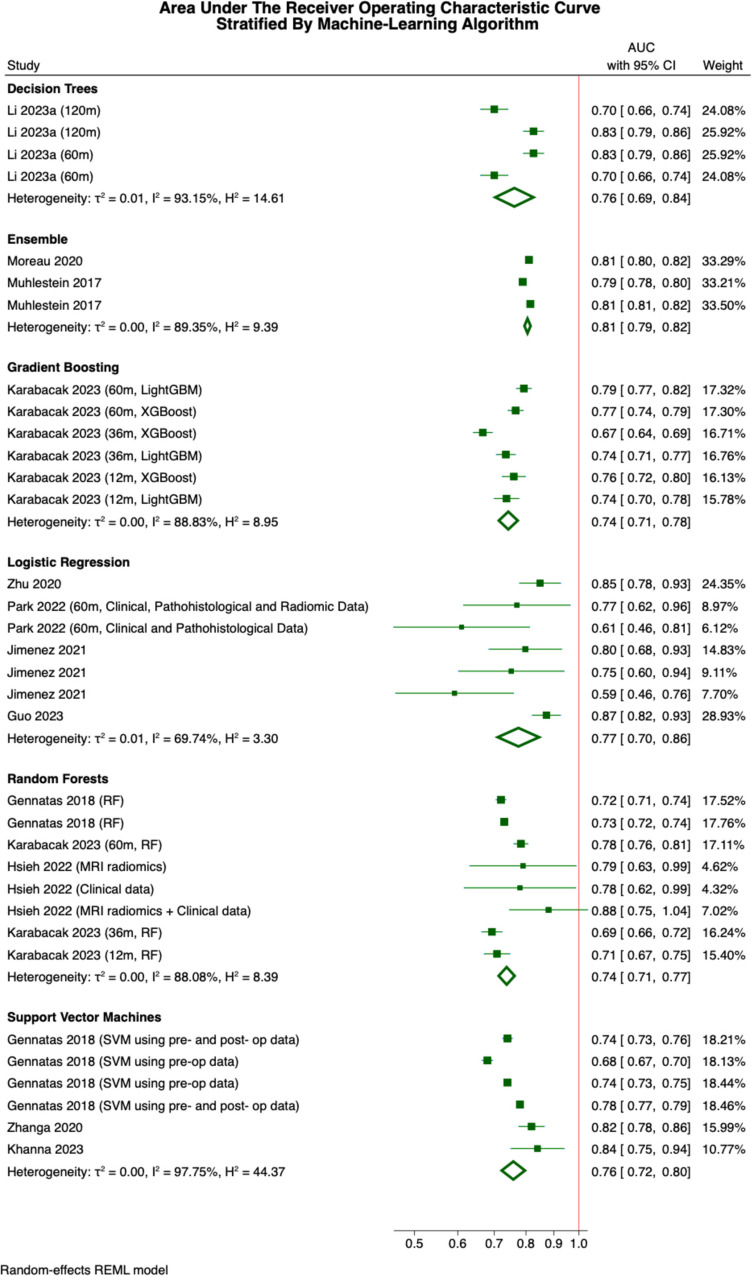
Area under the receiver operating characteristic curve stratified by the class of ML algorithm employed in outcome prediction. Weights are given with respect to each class. Where the same paper is included multiple times, each estimate is discriminated by the time course of outcome prediction in months, the ML algorithm employed or the modality of the input data. Note that LightGBM and XGBoost are two alternate gradient boosting algorithms

Meta-analyses were conducted to generate pooled estimates for the predictive accuracy of 6 classes of ML algorithms and for all ML algorithms in predicting each of 5 different post-operative outcomes. The pooled estimates for the accuracy of ML algorithms in predicting discharge disposition, length of hospital stay, overall survival, recurrence-free survival, and survival with neither recurrence nor progression were as follows: 0.79 (0.78–0.80), 0.81 (0.81–0.82), 0.73 (0.71–0.76), 0.74 (0.71–0.78) and 0.83 (0.74–0.93), respectively. Only the analyses of overall survival and recurrence-free survival incorporated data from more than two studies, and both suffered from a high degree of heterogeneity, I^2^ > 95% for both [12]. ML algorithms demonstrated superior accuracy in predicting survival without recurrence or progression compared to overall survival (*p* = 0.04). However, there was no significant difference in accuracy between predicting recurrence-free survival and either of the two outcomes. Regarding alternate ML algorithms, the AUC estimates for logistic regression, random forests and support vector machines incorporated data from more than two studies, as demonstrated by Fig. 3. The pooled estimates for the AUCs achieved with each class of ML algorithm were 0.77 (0.70–0.86), 0.74 (0.71–0.77) and 0.76 (0.72–0.80). No two of these classifiers differed significantly in terms of predictive accuracy as quantified by the area under the receiver operator characteristics curve (*p* > 0.05). However, the reliability of these estimates is limited by the moderate to high heterogeneity in the individual data points for each outcome (I^2^ values of 69.7%, 88.1% and 97.8%, respectively) [12]. Notably, our pooled estimate for the AUC of random forest classifiers did not differ significantly from that of simple decision tree classifiers; however, when this analysis was limited to models predicting overall survival, decision tree classifiers (both from a single paper) [20] significantly outperformed random forests of multiple decision trees (0.83 (0.80–0.85) compared to 0.73 (0.69–0.77), *p* < 0.00005). Rather than representing a superior predictive accuracy of decision trees, this observation could also be explained overfitting of the included random classifier models to their training data through excessive regularisation of hyperparameters.

### Multimodal data input

Published studies predominantly utilised either clinical variables (including tumour grade, demographics, comorbidities, and histopathology) or radiomic data in order to stratify the risk of each postoperative outcome, with a small minority of studies incorporating both modalities into the same model. Each of the 3 studies which directly compared the former to the latter found that a combined model yielded superior predictive accuracy: the addition of radiomics to a logistic regression model of clinical and histopathological data was found to improve the AUC for the prediction of 5-year recurrence-free survival from 0.61 (0.44–0.78) to 0.77 (0.60–0.94) [28]. Similarly, the addition of MRI radiomics to clinical data yielded an AUC of 0.880 (0.714–0.993) for the prediction of survival free from both recurrence and progression. Compared to a random forest model using either data modality individually, this represented a significant improvement in predictive accuracy [29]. Finally, the performance of a support vector machine model in predicting both 5-year overall survival and 5-year recurrence-free survival was found to benefit from the addition of postoperative data. When tumour grade, extent of resection and adjuvant radiotherapy were incorporated alongside preoperative clinical variables, the AUC reached 0.74 (0.73–0.76) and 0.78 (0.77–0.79), respectively, for the combined model compared to 0.68 (0.67–0.70) and 0.74 (0.73–0.75), respectively, for the exclusively preoperative model. [33]

### Ensemble classifiers

Five papers integrated multiple ML approaches into a single ensemble classifier [18, 19, 24, 26, 32, 43]; however, data availability prohibited all but 2 [24, 26] from being incorporated into our meta-analysis (Fig. 3). The first of these employed a combination of balanced logistic regression and random forests to predict 5-year overall survival with a sensitivity of 0.79 and specificity of 0.75, while the second utilised an ensemble of support vector machines, logistic regression and gradient boosting to predict postoperative discharge disposition [43]. The latter further developed this by incorporating Vowpal Wabbit (ML library) and stochastic gradient descent alongside the three aforementioned algorithms to generate a prediction for the length of inpatient admissions [26]. The former study achieved sensitivity of 0.755 (0.750–0.760) and specificity of 0.689 (0.679–0.698), while the latter exhibited a positive predictive value of 0.588 (0.586–0.590) and negative predictive value of 0.862 (0.860–0.864). Our meta-analysis found these ensemble models to significantly outperform both random forests and gradient boosting at the 99% confidence level (AUC of 0.81 (0.79–0.82) for ensemble models compared to 0.74 (0.71–0.77) for random forests and 0.74 (0.71–0.78) for gradient boosting). Ensemble classifiers showed no such advantage over decision trees, classification methods, or support vector machines in our meta-analysis at the 95% confidence level. However, 1 study demonstrated that an ensemble model combining support vector machines, classification methods, and random forests outperformed a single classifier alone (AUC of 0.78 and 0.71, respectively) [27]. It is important to note that 2 of the studies exploring ensemble classifiers used a leaderboard approach for algorithm selection. Therefore, these findings represent the best predictive accuracy among 26 and 34 algorithms, respectively, rather than an average AUC for a standard ensemble model. [26, 27]

### Meta-regression

**Fig. 4:**
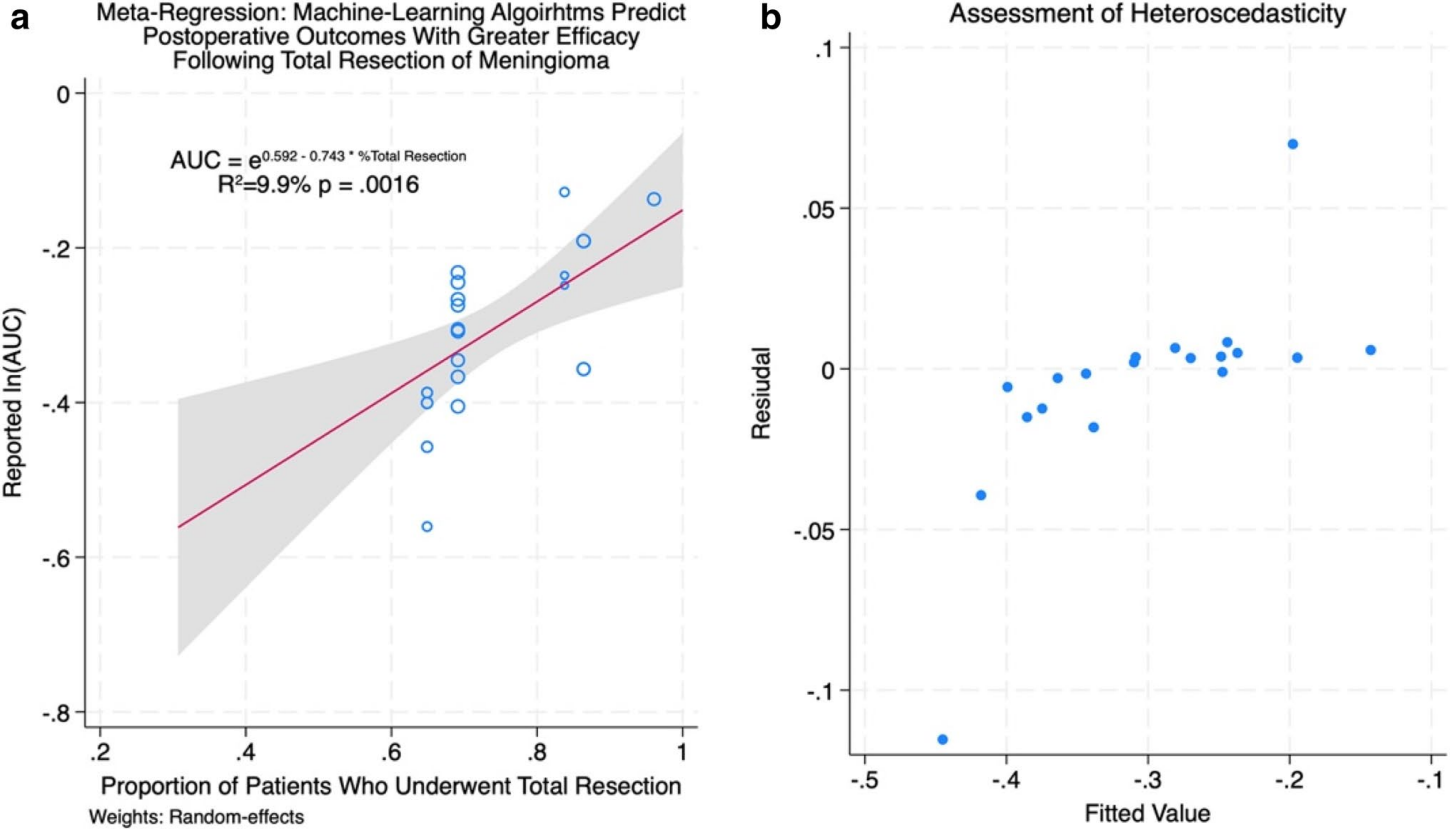
**a** Meta-regression % total resection. **b** assessment of heteroscedasticity

**Fig. 5:**
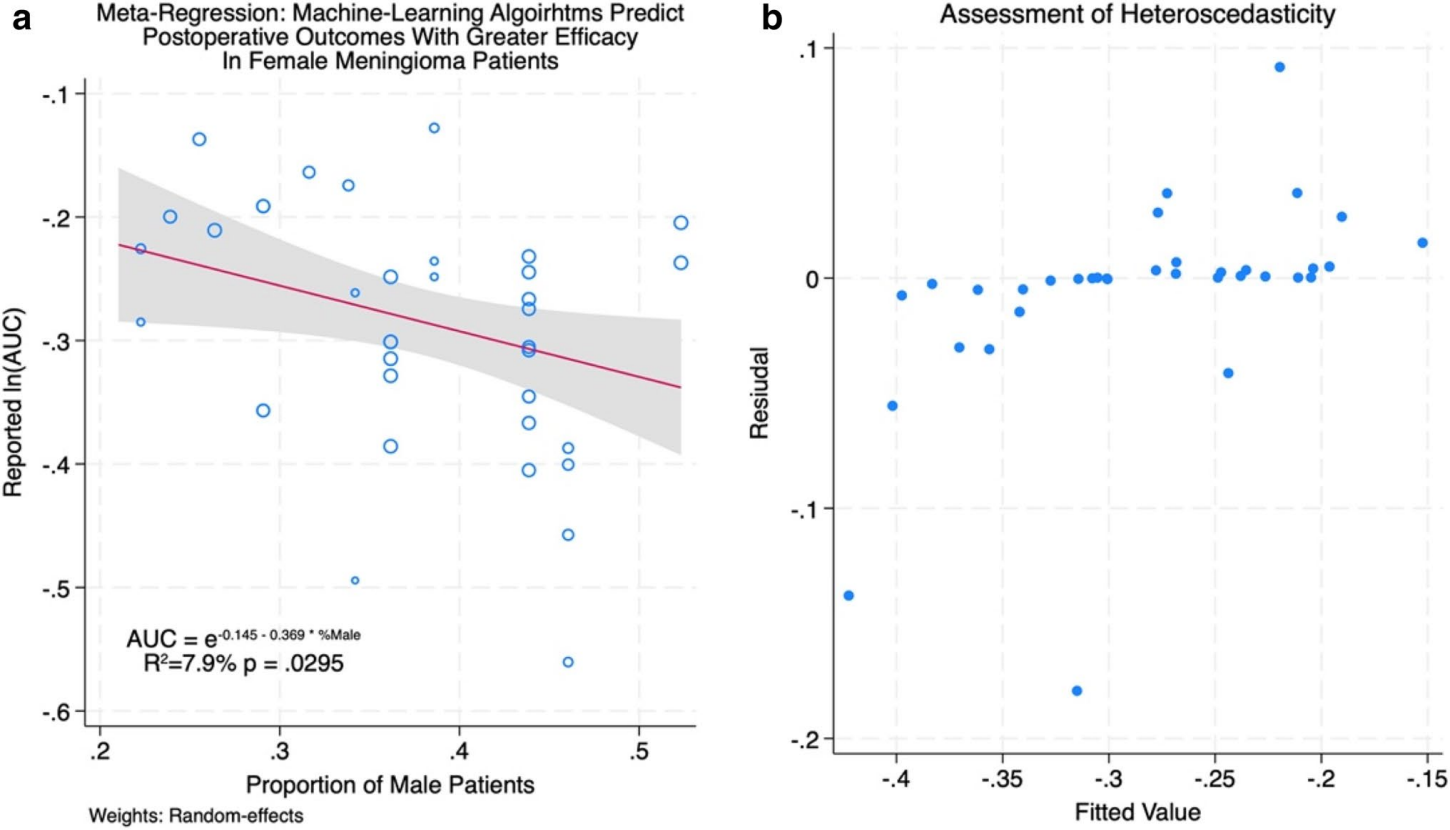
**a** Meta-regression % total resection. **b** meta-regression % total resection

**Fig. 6:**
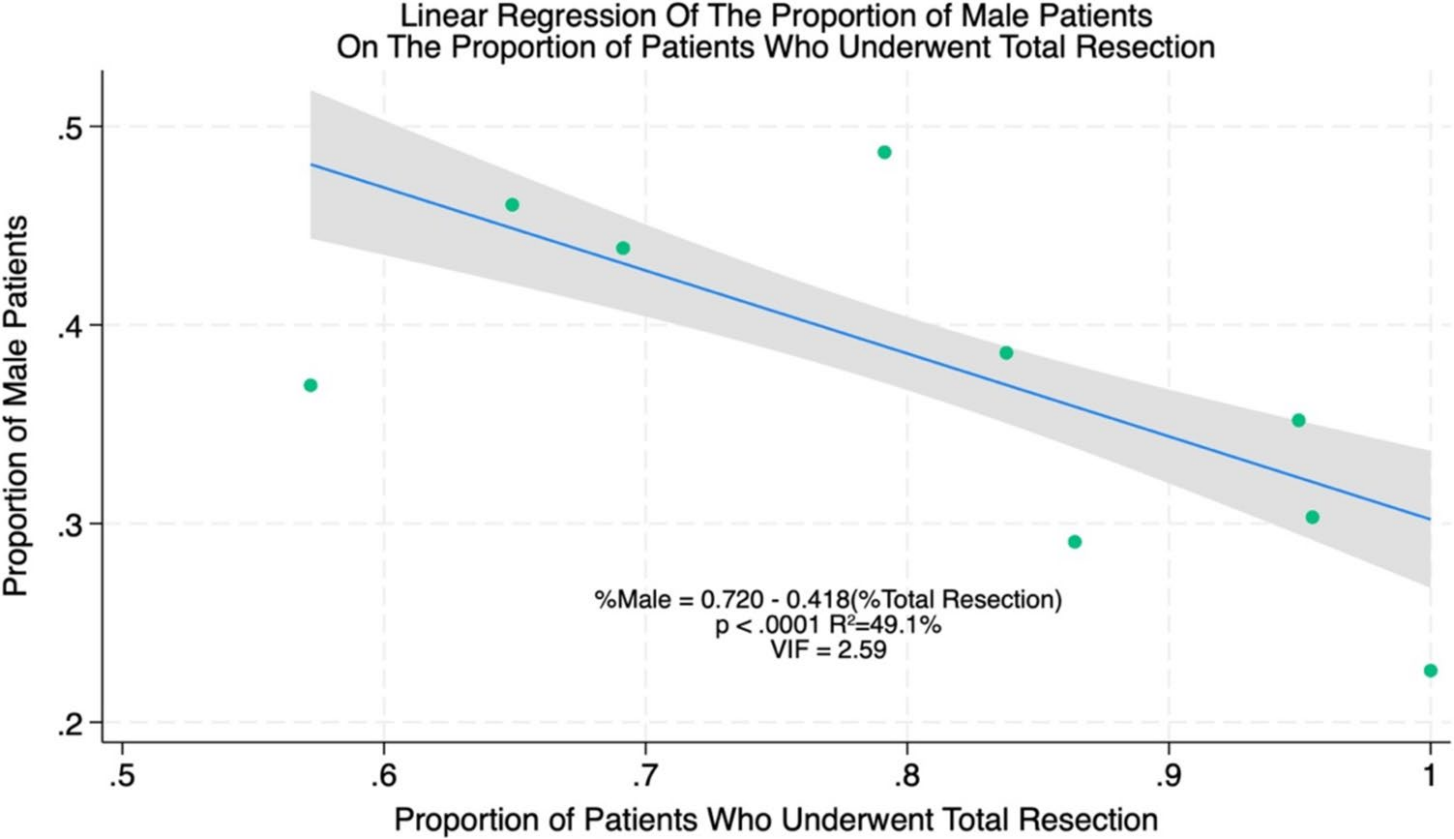
Auxillary linear regression

Results of the meta-regressions are presented in Figs. 4a and 5a (meta-regression % total resection and meta-regression male). The log-transformed AUC was found to have a significant correlation with the proportion of male patients (*p* = 0.0295) and with the proportion of patients who underwent total resection (*p* = 0.0016) across the studies included in our meta-analysis. These covariates were found to account for 7.9% and 9.9% of the variation in reported AUCs by different groups, while the mean age of meningioma patients was not significantly correlated with the AUC. These findings suggest that ML models may predict outcomes with superior efficacy in female patients and following total resection of meningioma compared to male patients or following subtotal resection. The homoscedasticity of both covariates against ln(AUC) was confirmed via visual inspection of the residual plots in Figs. 4b and 5b (meta-regression % total resection and meta-regression male), verifying the validity of the linear relationship. The auxiliary regression presented in Fig. 6 (auxiliary linear regression) demonstrates significant collinearity between these covariates (*p* = 0.0002), with higher proportions of male patients corresponding to lower rates of total resection, although the variance inflation factor was moderately low (VIF = 2.91) for these covariates. [2]

### Study quality assessments

Using the Risk of Bias in Non-randomized Studies—of Interventions (ROBINS-I) presented in Table 1 [35]**,** each study included in this review underwent a critical analysis and a final rating of ‘good’, ‘moderate’, or ‘poor’. Each study was critiqued for ‘selection bias’, ‘confounding’, ‘outcome reporting’, attrition bias’, and ‘overall risk of bias’, as per ROBINS-I criterion. A total 2/32 (6.25%) were assigned a rating of ‘good’, 4/32 (12.5%) were assigned a rating of ‘moderate’, and 26/32 (81.25%) were assigned a rating of ‘poor’.

**Table 1:** Risk Of Bias In Non-randomised Studies - of Interventions (ROBINS-I) of each included study. Good, low risk of bias; moderate, acceptable risk with some limitations; poor, significant risk of bias likely to affect outcomes

Study citation	Selection bias	Confounding	Outcome reporting	Attrition bias	Overall risk of bias	Quality
Burkhardt et al. [2]	Moderate	High	Moderate	Moderate	High	**Poor**
Cai et al. [3]	High	High	Moderate	Moderate	High	**Poor**
Chen et al. [4]	Moderate	Moderate	Moderate	Low	Moderate	**Moderate**
Chotai et al. [5]	Moderate	High	Moderate	Low	High	**Poor**
Deist et al. [6]	Low	Low	Low	Low	Low	**Good**
Escribano Mesa et al. [7]	High	High	Moderate	Low	High	**Poor**
Gennatas et al. [8]	Moderate	High	Moderate	Low	High	**Poor**
Guo et al. [10]	High	High	Moderate	Low	High	**Poor**
Hsieh et al. [11]	Moderate	High	Moderate	Low	High	**Poor**
Jimenez et al. [13]	Low	High	Moderate	Low	High	**Poor**
Jo et al. [14]	High	High	Moderate	Moderate	High	**Poor**
Karabacak et al. [15]	Moderate	High	Moderate	Low	High	**Poor**
Karri et al. [16]	Moderate	High	Moderate	Moderate	High	**Poor**
Khanna et al. [17]	Low	Moderate	Low	Low	Moderate	**Moderate**
Ko et al. [18]	High	High	Moderate	Low	High	**Poor**
Li et al. [19]	High	High	Moderate	Low	High	**Poor**
Li et al. [20]	High	High	Moderate	Low	High	**Poor**
Moreau et al. [24]	Moderate	High	Moderate	Moderate	High	**Poor**
Morin et al. [25]	High	High	Moderate	Low	High	**Poor**
Muhlestein et al. [26]	High	High	Moderate	Low	High	**Poor**
Muhlestein et al. [27]	High	High	Moderate	Low	High	**Poor**
Nguyen et al. [28]	Low	Moderate	Moderate	Low	Moderate	**Moderate**
Park et al. [30]	High	High	Moderate	Low	High	**Poor**
Sahm et al. [32]	High	High	Moderate	Moderate	High	**Poor**
Song et al. [34]	High	High	Moderate	Low	High	**Poor**
Wang et al. [37]	High	High	Moderate	Low	High	**Poor**
Yuan et al. [38]	High	High	Moderate	Low	High	**Poor**
Zador et al. [39]	Low	Moderate	Moderate	Low	Moderate	**Moderate**
Zhang et al. [41]	Low	Low	Low	Low	Low	**Good**
Zhang et al. [42]	High	High	Moderate	Low	High	**Poor**
Zhao et al. [44]	High	High	Moderate	Low	High	**Poor**
Zhu et al. [45]	High	High	Moderate	Low	High	**Poor**

## Discussion

### Summary of results

This systematic review and meta-analysis identified a total of 32 papers assessing the accuracy of ML algorithms in predicting post-operative outcomes in meningioma patients. To our knowledge, this is the first paper to generate pooled estimates for predictive accuracy metrics in meningioma patients with regards to postoperative outcomes. Our findings demonstrate a crucial gap in the literature assessing the utility and validity of ML algorithms: the absence of a direct comparison between the predictive accuracy of ML models compared to traditional methods. ML algorithms provide a novel solution to a perennial issue in neurosurgery: reliably predicting patient prognosis prior to surgical resection. Patients are understandably preoccupied with their likely prognosis but there has been, until recently, no reliable method to predict this before offering surgical resection. By harnessing both clinical and radiomic data, ML models should enable precise risk stratification and personalised treatment approaches in the coming years. However, studies published to date suffer from a paucity of direct comparisons with traditional methods.

A study published in 2023 compared traditional ML versus deep learning in meningioma classification, grading, outcome prediction and segmentation [22], and conducted a meta-analysis comparing ML to deep learning which yielded promising results in favour of ML. However, this study only compared ML to deep learning only and made no mention or comparison to the use of traditional protocols. Additionally, only one of the studies included in that review directly compared ML to deep learning. In our study, meta-analysis was conducted for the areas under the receiver operating characteristic curve (AUCs) reported by each paper so long as data availability allowed the variance therein to be calculated. This was repeated for each of the five most frequently reported postoperative outcomes and for the most frequently reported ML methods to generate overall estimates for their respective efficacies. ML algorithms were more accurate in predicting survival without recurrence or progression than overall survival, but not significantly different in predicting recurrence-free survival.

This review provides evidence that ML algorithms may perform better in cohorts with a high proportion of female patients, a finding which may be attributed to meningiomas occurring more than twice as frequently in adult females [1, 20, 30, 37, 45] and thus, training data are likely to be highly biased towards the outcomes of female patients. Furthermore, meningiomas in female patients are typically low-grade, while those in males are often malignant [1], which may be related to a protective effect of higher serum testosterone concentrations [1, 36]. Indeed, men with prostate cancer who undergo androgen deprivation therapy show increased meningioma growth; additionally, meningioma biopsies have been found to express luteinizing hormone-releasing hormone receptors, suggesting that high testosterone levels may be protective against progression and recurrence of meningioma [1, 36]. These findings highlight that without validation of predictive models in a novel dataset, there is a tendency to overfitting towards the specific characteristics or idiosyncrasies of the training set.

In addition to ML, pathology-based predictors such as methylation arrays show promise in stratifying meningioma patients post-resection, particularly in identifying aggressive subtypes that may not be detected through traditional histopathology [36]. In our review, 3/32 studies [19, 28, 32] utilised WHO grading in their ML algorithms, none used methylation information. However, interest in using methylation analysis as an additional classification tool is a relatively recent development and has yet to be widely adopted in all centres, with varying protocols for when it is performed. While methylation arrays provide valuable insights into tumour biology, ML models are better in integrating these molecular data with clinical and imaging variables to offer more comprehensive and personalised predictions.

Meta-regressions were conducted using log-transformed AUCs against three covariates (mean age, sex ratio, and proportion of patients with total tumour resection) to identify potential confounding variables. This analysis revealed significant correlations between ln(AUC) and both the proportion of male patients (*p* = 0.0295) as well as the proportion of patients with total resection (*p* = 0.0016). These findings could indicate that ML models predict outcomes more effectively in female patients or following total tumour resection compared to males or following subtotal resection; however, as stated above, this may in fact represent a bias in the training data rather than inherent differences in the risk of outcomes in male patients. Similarly, superior predictive accuracy is to be expected in studies with high rates of total resection as this would cause their cohorts to be more homogeneous, i.e. degree of resection would no longer contribute to variance in the outcomes of interest in the majority of patients assessed, with < 20% of patients differing in terms of their Simpson grade in the studies with the most accurate ML models. Auxiliary regression demonstrated significant collinearity between the sex ratio and rate of total resection in included studies, thus, our data cannot conclude with any certainty which of these two covariates, if either, is responsible for variation in predictive accuracy or whether they are simply correlated.

### Limitations, risk of bias, and strengths

Our results demonstrate that ML algorithms can predict postoperative outcomes with accuracy equivalent to logistic regression models; however, most studies failed to include a comparison with conventional algorithms. Future papers should aim to directly compare these two approaches to determine whether the integration of ML algorithms into routine clinical practice can be justified. This may be performed through data analysis of the raw data that could be collected from the various papers included. 20/32 papers could not be included in the meta-analysis due to their failure to report comparable metrics such as the AUC, sensitivity, specificity or other related figures. Those that were included often failed to compare the efficacy of different ML algorithms and as a result only provided a point estimate for the efficacy of a specific model in predicting the outcomes of a select cohort of patients. Similarly, studies rarely reported data for multiple outcomes of interest and instead tended to focus on overall survival or recurrence-free survival; as a result, our comparison of AUCs for different outcomes may have limited validity due to the different populations being examined for each outcome. While informative for comparison between ML methods, estimates for the AUC lack the clinical applicability of metrics like sensitivity and false-positive rate, which are more intelligible for patients. Furthermore, while ML models can predict outcomes with superior efficacy in female patients following total resection of meningioma compared to male patients who underwent subtotal resection, the exact reason is not known. It may be attributed to a higher proportion of females included, as female tumours are usually benign while males’ are frequently high grade, which would lead to a greater number of resections indicating a that the data is over-fitted to the specific characteristics of the training set [1, 35]. Finally, the Core Outcome Sets for Meningioma in Clinical Studies (COSMIC) development, though a significant step for standardising meningioma studies, was not utilised by the included studies to inform their selection of outcomes.

The high-degree of heterogeneity (I^2^ > 75%) in the reported AUCs for overall survival and recurrence-free survival challenges the validity of our findings further. This level of heterogeneity reflects the wide variety of ML algorithms used by the studies in these analyses, which we have already shown to differ significantly in their efficacies. A high degree of heterogeneity suggest underlying data may differ too significantly between studies to draw reliable conclusions from pooled estimates. Future studies should aim to try and select more homogeneous datasets or exploring reasons for variability more rigorously.

Populations used to train and validate these algorithms differed notably between included papers, including the sex ratio and rates of total resection which we show to have been correlated with the ln(AUC) on meta-regression. The results of these meta-regressions, while significant, cannot determine with any reasonable certainty which of the two covariates, if either, are directly related to the efficacy of ML algorithms, or whether they are simply correlated with another as-of-yet unconsidered covariate. Only five papers discussed specific complication rates, and a lack of standardised reporting impeded comparative analysis. Also, none of the included papers made any reference to the cost effectiveness of ML classifiers, nor was there any reference to the financial or temporal investments required for a sufficient proportion of the multi-disciplinary team to be trained to use and interpret ML algorithms. Accordingly, it is difficult to determine their utility in a real-world setting, especially with respect to low-to-middle-income countries [24].

**Fig. 7:**
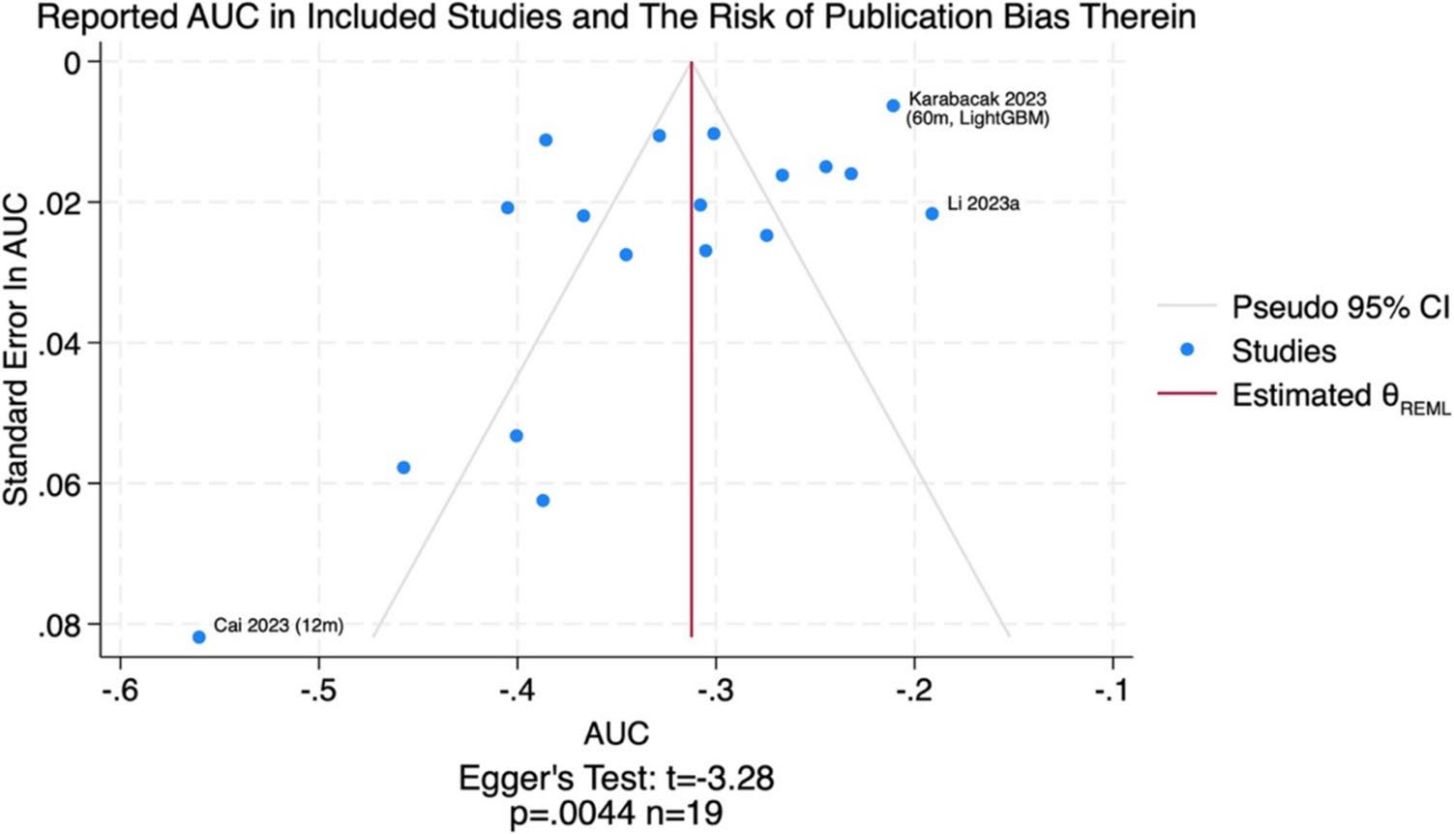
Funnel plot demonstrating the high risk of publication bias

Funnel plots (Fig. 7) exhibited an apparent bias towards the publication of studies which report lower values of AUC, exemplified by outliers such as one paper [3] which reported an AUC of 0.571 (0.481–0.663) in the prediction of 1-year overall survival. The Egger’s test statistic was also suggestive of significant publication bias in the reported AUCs: t = −3.28 (*p* = 0.0044).

## Conclusion

Integration of ML algorithms in predicting postoperative outcomes for meningioma patients presents a promising avenue for advancing medical decision-making. Our meta-analysis highlights the potential of ML algorithms, particularly logistic regressions, random forests, and support vector machines, in accurately predicting key outcomes. These algorithms have the potential to enhance prognostic assessments and personalise treatment strategies for managing meningioma. However, interest in additional predictors like methylation arrays is more recent and inconsistently applied across centres, which may limit their current role in comprehensive predictive models. Whether they will soon overtake traditional protocols for post-operative patient outcome prediction remains unclear. Traditional predictive methods, thoroughly researched and validated, serve as a robust foundation for clinical decision-making in meningioma management. In contrast, the literature on ML and its constituents regarding meningioma management is still in its infancy and so further research and development will be necessary, especially in refining methodologies and addressing biases, before ML implementation in clinical settings becomes feasible. By strategically incorporating these cutting-edge technologies and upholding the proven standards of existing treatment protocols, healthcare professionals can fully capitalise on the capability of ML algorithms to enhance patient care and optimise treatment strategies.

## Supplementary Information

Below is the link to the electronic supplementary material.

Supplementary Table 1 Summary of included studies and extracted data (XLSX 47.9 KB)

## Data Availability

No datasets were generated or analysed during the current study.
